# Fibroblast Growth Factor 21 Modulates Microglial Polarization That Attenuates Neurodegeneration in Mice and Cellular Models of Parkinson's Disease

**DOI:** 10.3389/fnagi.2021.778527

**Published:** 2021-12-22

**Authors:** Changwei Yang, Wuqiong Wang, Pengxi Deng, Chen Li, Liangcai Zhao, Hongchang Gao

**Affiliations:** ^1^School of Pharmaceutical Science, Institute of Metabonomics & Medical NMR, Wenzhou Medical University, Wenzhou, China; ^2^School of Public Health, Fujian Medical University, Fuzhou, China

**Keywords:** dopaminergic neurodegeneration, fibroblast growth factor, microglia phenotype, neuroinflammation, sirtuin 1, cognitive decline

## Abstract

Microglial polarization and the subsequent neuroinflammatory response were identified as key contributors to the progress of Parkinson's disease (PD). Researchers have shown that fibroblast growth factor 21 (FGF21) plays multiple biological functions, including anti-inflammation and neuroprotection. However, the knowledge of FGF21 on microglial polarization in PD *in vivo* is far from completion. In this study, both *in vivo* and *in vitro* models were used to investigate whether FGF21 enhances the brain function by modulating microglial polarization in PD. The protective effects of FGF21 *in vivo* were conducted using 1-methyl-4-phenyl-1,2,3,6-tetrahydropyridine-induced PD mice model alongside intraperitoneally received FGF21. A behavioral test battery and tyrosine hydroxylase (TH) immunohistochemistry were conducted to evaluate the neuronal function and nigrostriatal tract integrity. Immunofluorescence assay and Western blot were used to examine M1/M2 microglial polarization. Then, a microglia-neuron co-culture system was adopted *in vitro* to identify the underlying molecular mechanisms of FGF21. The results showed that FGF21 significantly alleviated motor and cognitive impairment in mice with PD. FGF21 also protected TH-positive neuron cells in the striatum and midbrain. Mechanistically, FGF21 suppressed M1 microglial polarization and the subsequent mRNA expression of pro-inflammatory factors while promoting M2 microglial polarization with increasing anti-inflammatory factors in mice with PD. Furthermore, sirtuin 1 (SIRT1) and the nuclear factor-kappa B (NF-κB) pathway were involved in the FGF21-induced M2 microglial polarization. Conversely, SIRT1 inhibitor EX527 significantly prevented both the FGF21-induced SIRT1 expression and M2 microglial polarization. Moreover, FGF21 pretreatment of microglia significantly prevented neuronal cell apoptosis in a microglia-neuron co-culture system. In conclusion, our data demonstrate that FGF21 exerted its protective effects in the pathology of PD through SIRT1/NF-κB pathway-mediated microglial polarization. Given the safety record of human clinical trials, FGF21 could be a promising therapy for clinical trials to ameliorate motor and nonmotor deficits in patients with PD.

## Introduction

Parkinson's disease (PD) is an age-related neurodegenerative disorder hallmarked by chronic degeneration of dopaminergic neurons in the midbrain (Rai and Singh, 2020; Rai et al., 2021). The exact mechanisms underlying the process in PD are not well-understood, and new early detection markers and therapeutic targets are urgently needed (Postuma et al., 2015; Rietdijk et al., 2017). Thus, increasing the survival of dopaminergic neurons or alleviating neuroinflammation may represent new treatment strategies.

Even though PD has been historically studied within the dopaminergic neurons, other cells, including microglial and astrocytic dysfunctions, also contribute to the progression of dopaminergic neuron degeneration in PD (Yun et al., 2018). In fact, increasing evidence strongly suggested that microglia-mediated inflammation may play critical roles in the neurodegenerative process in PD (Liddelow et al., 2017). More recent studies showed that neuroinflammation and associated “reactive” microglia could impart changes to the neural world around them, thus contributing to neurological illnesses in PD (Qin et al., 2021). Microglia has two phenotypes of M1 and M2, which show distinctive phenotypes that serve different functions (Tang and Le, 2016). Pro-inflammatory M1 microglia, induced by a persistent stimulus (e.g., misfolded alpha-synuclein in PD), produces a significant number of nitric oxide (NO) and pro-inflammatory cytokines, such as interleukin 1beta (IL-1β) and tumor necrosis factor (TNF-α), and these neurotoxic factors could cause neuronal damage in PD (Song et al., 2018). In contrast, anti-inflammatory M2 microglia exerts neuroprotective effects by producing neurotrophic factors, thereby promoting neuronal repair (Park et al., 2016). Although the molecular mechanism underlying microglial polarization in PD remains unclear, accumulating evidence showed that abnormal microglial polarization could contribute to the preferential damage of dopaminergic neurons, thus consistently involving in PD pathogenesis (L'Episcopo et al., 2018). Hence, the inhibition of microglia-mediated inflammation through modulating M1/M2 polarization may represent an effective way to relieve behavior dysfunction in PD.

Fibroblast growth factor 21 (FGF21) is a classic metabolic regulator that plays a critical role in glucose and lipid metabolism through fibroblast growth factor signaling in patients with obesity and animal studies (Gaich et al., 2013; Woo et al., 2013). Recently, FGF21 has received great interest as it could also be involved in aging (Yu et al., 2015), dementia (Chen et al., 2019), and obesity-induced cognitive impairment (Sa-Nguanmoo et al., 2018). The relevance of FGF21 to brain health is supported by the findings that FGF21 receptors were also expressed in the central nervous system (Chadashvili and Peterson, 2006), and the results have shown that circulating FGF21 could cross the blood–brain barrier (Hsuchou et al., 2007). More importantly, previous studies indicate that injecting intraperitoneally FGF21 into mice can significantly attenuate neurodegeneration in mice with diabetes (Kang et al., 2020) and obesity through metabolic modulation and anti-proinflammatory effects (Wang et al., 2018). FGF21 has also been found to protect against neurotoxicity induced by amyloid-β (Mona et al., 2018) and MPP^+^ in dopaminergic neurons (Fang et al., 2020). Moreover, FGF21 increases mitochondrial efficacy in human dopaminergic neurons *via* the NAD+-dependent deacetylase sirtuin 1 (SIRT1) (Mäkelä et al., 2014). Notably, FGF21 could be considered a promising target in alleviating neurodegeneration in PD, and the knowledge of FGF21 on microglial polarization in PD *in vivo* is far from completion.

Whether FGF21 can acutely enhance brain function and/or modulate microglial polarization is a gap in our knowledge of its therapeutic potential. In this study, we verified the hypothesis that FGF21 supplementation enhances brain function through anti-inflammatory properties mediated by the modulation of microglial polarization both *in viv*o and *in vitro*. We showed that FGF21 protects against the loss of dopaminergic neurons and cognitive and motor deficits in the 1-methyl-4-phenyl-1,2,3,6-tetrahydropyridine (MPTP)-induced PD mice. Our results also revealed that the neuroprotective effects of FGF21 in PD seem to mediate by M2 microglial polarization through activation of SIRT1/nuclear factor-kappa B (NF-κB) signaling.

## Materials and Methods

### Animals and Treatments

Male C57BL/6 mice (6 weeks old) were purchased from SLAC Laboratory Animal Co. Ltd. (Shanghai, China) and housed in the experimental animal center of Wenzhou Medical University. After acclimating to the animal facility for 1 week, the mice were randomly divided into the following groups: control (CON), PD, and FGF21 groups. Since circulating FGF21 has been demonstrated to cross the blood–brain barrier (Hsuchou et al., 2007), FGF21 treatment was performed by intraperitoneal injection (i.p.) (Li et al., 2019). Mice in the FGF21 group were treated with FGF21 (1.5 mg/kg; i.p. School of Pharmaceutical Science, Wenzhou Medical University) daily for 7 days, based on an established dose regimen of FGF21 in mice (Wang et al., 2018). Mice in PD and FGF21 groups were received with MPTP-HCL (30 mg/kg, i.p., M086, Sigma-Aldrich, USA) once a day for 5 consecutive days, according to our previous reports (Lu et al., 2018). Mice in the CON group received an equal volume of normal saline. Food and water were available during the whole experimental process. All experiments were performed in accordance and approved by the Institutional Animal Care and Use Committee of Wenzhou Medical University (document number: wydw2018180).

### Assessment of Motor Deficits and Cognitive Function

Pole test, rotarod test, open field test, Y-maze, and Morris water maze were carried out to evaluate the motor and non-motor functions of mice. All behavioral tests were assessed in the order listed below over 14 days to reduce the carryover effects.

#### Pole Test

Pole test has been widely used test to quantify motor functions (Brooks and Dunnett, 2009). Mice were trained to complete the task 3 days before testing. Briefly, mice were placed on the top of a vertical pole with their head upward. On the test day, the total time to descend to the floor (T total) and total time to turn down were recorded for 3 trials.

### Rotarod Test

An accelerating rotarod apparatus (model BW-ZH600; Shanghai, China) was used for the rotarod test as described previously (Brooks and Dunnett, 2009). Mice were placed on a rotating drum and tested at the speed of 30 rpm for a total of 10 min and latency to fall was recorded.

### Open Field Test

Mice were placed in the center of the apparatus lightly, allowing it to explore freely for 5 min. After each test, 75% alcohol solution was used to remove the odor clues in the interior of the box. Animal activities were monitored using an animal behavior analysis system (Shanghai, China). Behavioral changes were included total distance traveled, and central distance and peripheral distance were calculated.

### Y-Maze and Morris Water Maze

The cognitive function of the mice was assessed in the Y-maze and Morris water maze. As for the Y-maze, mice were placed at one end of the arm and tested for 5 min. The percentage of alternation was defined as the [total number of alternations/(total number of entries – 2)] ×100%. In the Morris water maze, the pool was divided into four quadrants with equal size. In the target guardant, a circular platform was placed and submerged 1 cm below. First, in the training session, the mice received consecutive training, 4 times a day, for 5 days. The mice were put into the pool with their face toward the wall and allowed to swim freely until they reached the escape platform. If the mice did not reach the platform within 65 s, it was gently guided to the escape platform and remained for 15 s. Total distance and latency to the platform were recorded. Second, in the test session, the platform was removed, and the mice were allowed to swim freely for 65 s. The number of crossing places of platform, distance, and time spent in the target quadrant were recorded.

### Immunohistochemistry

Mice (*n* = 3 for each group) were anesthetized and then perfused with 4% paraformaldehyde for 30 min. The whole brain was rapidly dissected and processed for the paraffin section. The paraffin-embedded sections were then deparaffinized and rehydrated, followed by incubation with primary antibody to tyrosine hydroxylase (TH; Cell Signaling, USA) and ionized calcium-binding adaptor molecule-1 (Iba-1) (1:300, 019-19741, Wako Japan) at 4°C for 24 h. After incubated with a second antibody (Alexa 488-labeled secondary antibodies, 1:500, ab150077, Abcam USA, or a Goat Anti-Rabbit IgG H&L), followed by DAPI nuclei staining (0100-20, SBA), the immunoreactivity was visualized with a Nikon ECLIPSE 80i (Nikon, Japan). Quantification of TH and Iba-1 staining densities was measured using the ImageJ software.

### Neuronal Apoptosis

The TdT-mediated dUTP nick-end labeling (TUNEL) assay was performed using a commercial TUNEL kit (C1086, Beyotime, China) in the paraffin-embedded brain sections, which was used to assess the neuronal apoptosis according to the instructions of manufacturer. Briefly, after deparaffinized and rehydrated, the brain sections were treated with proteinase K (20 μg/ml, Apoptosis Detection Kit; Merck Millipore, USA) for 30 min at 37°C; then, the brain sections were then incubated with a TUNEL reaction mixture for 60 min at 37°C. All the reactions were performed in the dark. Sections were visualized and captured at 20 × by using a Nikon ECLIPSE 80i (Nikon, Japan).

### Real-Time PCR Analysis

Total RNA extraction from the midbrain and striatum was carried out using a standard TRlzol reagent (Invitrogen, Carlsbad, CA, USA), after converting into cDNA with Prime Script™ RT reagent Kit (Takara, RR037A, Japan), according to our previous report (Yang et al., 2014). cDNA, corresponding to 50 ng total RNA, was served as a template in a 10 μl reaction mixture for the targeted mRNA expression. Real-time PCR amplification was performed on a Bio-Rad quantitative PCR system (CFX ConnectTM Real-Time System, Bio-Rad, CA, USA) using SYBR Green PCR Master Mix Kit (Applied Biosystems, Carlsbad, CA, USA). GAPDH mRNA level was used to analyze the relative mRNA expression of the target genes. The detailed real-time PCR reaction conditions and primer sequences are given in Table 1.

**Table 1:** Primers used for quantification of targeted genes in Quantitative real-time PCR.

**Gene name**	**Primer**	**Primer sequence (5^′^-3^′^)**
GAPDH	Forward	AGGTCGGTGTGAACGGATTTG
	Reverse	TGTAGACCATGTAGTTGAGGTCA
IL-1β	Forward	TGCCACCTTTTGACAGTGATG
	Reverse	AAGGTCCACGGGAAAGACAC
TNF-α	Forward	CCCTCACACTCAGATCATCTTCT
	Reverse	GCTACGACGTGGGCTACAG
IL-10	Forward	GCTCTTACTGACTGGCATGAG
	Reverse	CGCAGCTCTAGGAGCATGTG
Arg-1	Forward	CTCCAAGCCAAAGTCCTTAGAG
	Reverse	AGGAGCTGTCATTAGGGACATC
CD206	Forward	CTCTGTTCAGCTATTGGACGC
	Reverse	CGGAATTTCTGGGATTCAGCTTC
CD206	Forward	TGTCTGATCTTGCTAGGACCG
	Reverse	GAGAGTAACGGCCTTTTTGTGA
CD163	Forward	ATGGGTGGACACAGAATGGTT
	Reverse	CAGGAGCGTTAGTGACAGCAG

### Western Blot Analysis

The detailed protein extraction was described elsewhere (Yang et al., 2019). In brief, the total protein level was quantified using a protein assay kit (Bio-Rad, USA). Equivalent amount of proteins were separated by sodium dodecyl sulfate-polyacrylamide gel electrophoresis (SDS-PAGE), followed by being transferred to polyvinylidene fluoride (PVDF) membrane. The membranes were incubated with primary antibodies, including NF-κB and Phospho-NF-κB, SIRT1, Bax and Bcl-2, cleaved caspase-3 (1:1,000, Cell Signaling Technology, Danvers, MA, USA), inducible nitric oxide synthase (iNOS), IL-10 (1:1,000, Abcam, Cambridge, UK), and GAPDH (1:1,000, T0004-HRP, Affinity), at 4°C overnight, followed by HRP-conjugated secondary antibodies (1:5,000, ab6721 or ab6789, Abcam, USA) for 1 h at 25°C. Quantification of the protein bands was carried out by using the Image Analyses Software (ChemiDoc™ MP Imaging System Bio-Rad, USA).

### Cell Culture and Treatments

BV2 cells were cultured in high-glucose DMEM containing 10% fetal bovine serum and additional 1% penicillin/streptomycin. SH-SY5Y cells were cultured in DMEM/F-12 containing 15% FBS. To explore the regulation effect of FGF21 on microglia cell polarization, cells were treated with lipopolysaccharide (LPS) (100 μg/ml) with or without FGF21 for 6 h. To verify the role of SIRT1 in FGF21 regulating microglia cell polarization, cells were pretreated with SIRT1 inhibitor EX527 20 μM for 6 h before FGF21 and LPS treatment. The conditioned medium from the LPS-treated cells (LPS-CM) with either PBS or FGF21 treatment was applied to SH-SY5Y cells and incubated for 24 h for the neuronal cell death assay. Besides, nitric oxide (NO) assay in the conditioned medium was evaluated using the Griess test (Liu et al., 2005).

### Cell Viability

Cell viability was determined by a modified Cell Counting Kit-8 (CK04, Dojindo, Japan), according to the instructions of the manufacturer. After being seeded on a 95-well-plate, the SH-SY5Y cells were incubated with LPS, LPS-CM, FGF21, and FGF21-CM for 24 h. In addition, Hoechst 33342 (C0030, Solarbio, China) staining was conducted to verify the cell apoptosis. After incubated with LPS, LPS-CM, FGF21, and FGF21-CM for 24 h, SH-SY5Y cells were seeded on coverslips in a 24-well-plate. The coverslips were removed for Hoechst staining according to the instructions.

### Immunocytochemistry

To detect the subcellular distribution of the NF-kB p65 subunit, BV2 cells were seeded into a 24-well-plate and treated with LPS in the absence/presence of FGF21 for 6 h. Then, the cells were fixed with 4% paraformaldehyde. After permeabilization, cells were incubated with monoclonal rabbit anti-mouse NF-κB (p65) (1:200, 8242, Cell Signaling Technology, USA) at 4°C overnight, followed by incubation with the second antibody (Goat Anti-Rabbit IgG H&L; Alexa Fluor® 488, 1:500, ab150077, Abcam) and DAPI nuclei staining (0100-20, SBA), and then the immunoreactivity was visualized using a Nikon ECLIPSE 80i (Nikon, Japan).

The microtubule-associated protein 2 (MAP-2) staining was used to quantify the neuronal cell viability and membrane integrity. Briefly, SH-SY5Y cells were seeded into a 24-well-plate and then incubated with PBS, LPS-CM, and FGF21-CM for 24 h; 4% paraformaldehyde incubation for 20 min was used to fix the cells at 37°C. After permeabilization, cells were incubated with monoclonal rabbit anti-human MAP-2 (1:200, #8707S, Cell Signaling Technology) at 4°C overnight, followed by incubation with the second antibody (Goat Anti-Rabbit IgG, Alexa Fluor®, 488, 1:500, ab150077, Abcam, USA) and DAPI (0100-20, SBA) nuclei staining. The immunoreactivity was visualized using a Nikon ECLIPSE 80i (Nikon, Japan).

### Statistical Methods

Statistical analysis was conducted using SPSS for Windows 19 Inc., Chicago. One-way ANOVA analysis followed by an LSD multiple comparison test was used to identify the significance of group differences for normally distributed data. Data are presented as means and standard errors, and a significant level of *P* <0.05 was used.

## Results

### FGF21 Prevented Both Motor and Cognitive Deficits in MPTP-Lesioned Mice

**Figure 1:**
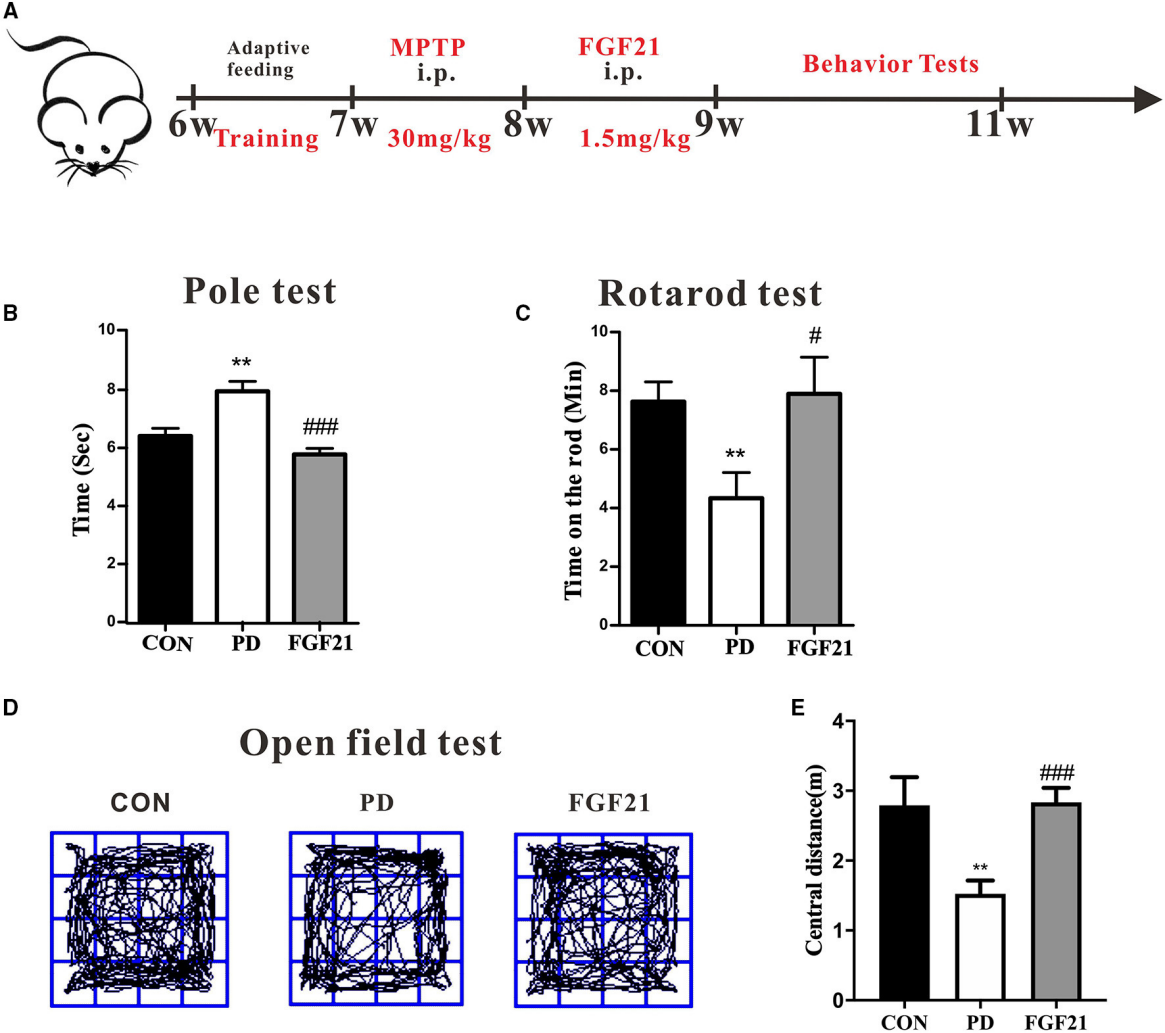
Fibroblast growth factor 21 (FGF21) treatment prevented the motor deficits of mice with PD. **(A)** Schematic of the experimental procedure. **(B)** Time to descend pole. **(C)** Rotarod test. **(D)** Track of mice in open field test. **(E)** Distance traveled in the center for 5 min. Data are mean ± s.e.m.; *n* = 6–8 for each group. One-way ANOVA was conducted for statistical analysis followed by an LSD multiple comparison test. **P < 0.01 vs. control (CON) group; ^#^*P* < 0.05, ^###^*P* < 0.001 vs. 1-methyl-4-phenyl-1,2,3,6-tetrahydropyridine (MPTP)-injected mice.

Motor dysfunction is a predominant deficit in mice with PD and is linked with clinical symptoms of patients with PD. To test the therapeutic potential of FGF21, we administered it intraperitoneally (i.p.) for 1 week after the injection of MPTP. The pole test, rotarod test, and open field test were conducted to monitor the motor function (Figure 1A). In the pole test, there is a marked increase in the total time to descend to the floor in mice with PD, which is reduced by FGF21 treatment (*P* <0.001, Figure 1B). FGF21 also significantly reduces the behavior deficits elicited by MPTP injection as measured by the rotarod test (*P* < 0.05, Figure 1C). In the open field test, mice with PD tended to spend less time in exploring the central part of the open field arena, and FGF21 treatment significantly increased the spontaneous activity in the central part of the arena (*P* < 0.001, Figures 1D,E).

**Figure 2:**
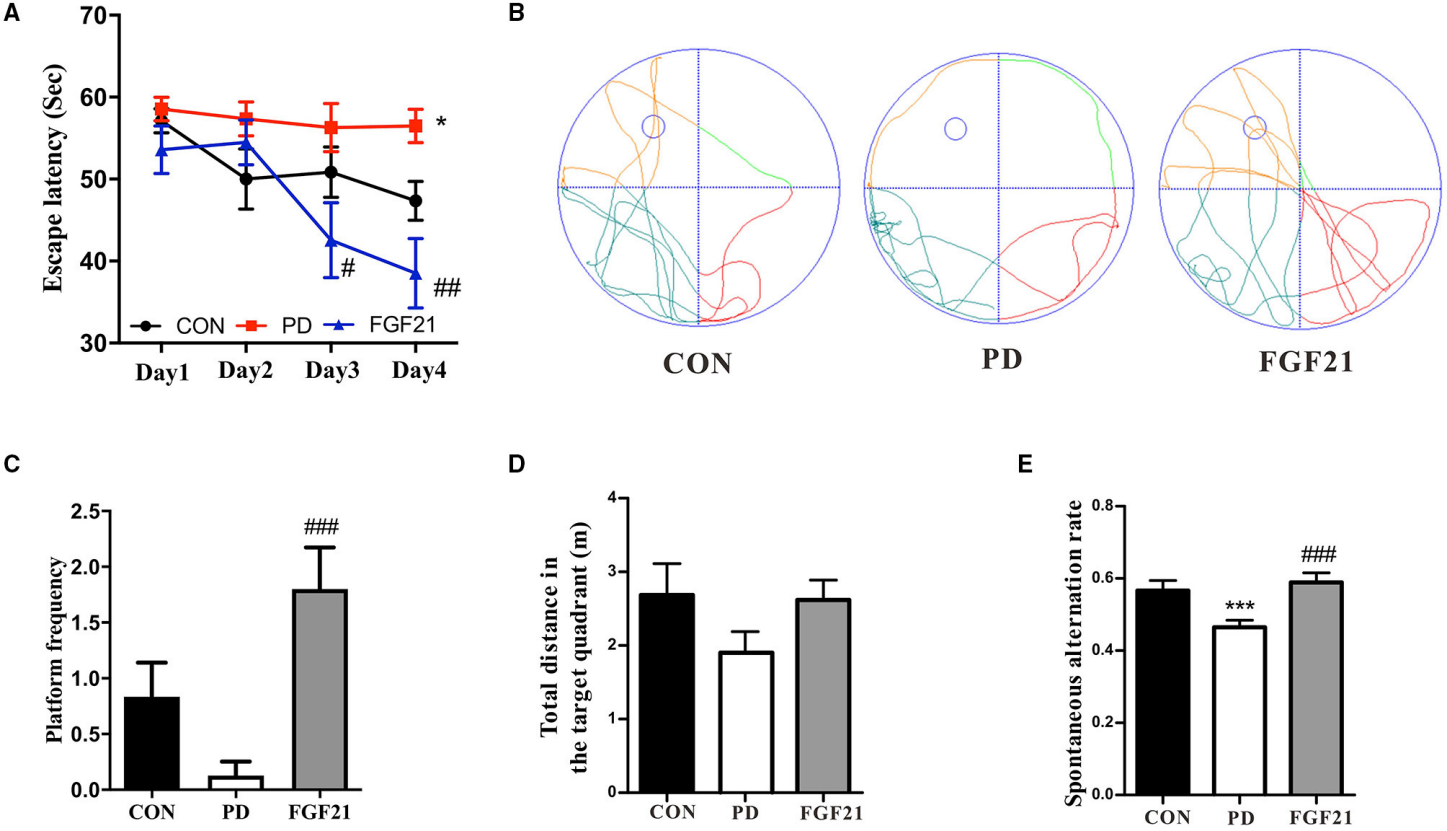
FGF21 treatment prevented the cognitive deficits of mice with PD. **(A)** Escape latency during 4 training days. **(B)** Representative swim paths. **(C)** Distance in the target quadrant. **(D)** Number of crossing the place of the platform. **(E)** Spontaneous alteration in the Y-maze. Data are mean ± s.e.m.; *n* = 6–8 for each group. A one-way ANOVA was used for statistical analysis followed by an LSD multiple comparison test. *P < 0.05, ***P < 0.001 vs. CON group; ^#^*P* < 0.05, ^##^*P* < 0.01, ^###^*P* < 0.001 vs. MPTP-injected mice.

We then tested whether FGF21 counters cognitive deficits in MPTP-lesioned mice in the Morris water maze and Y-maze. In the water maze, MPTP injection resulted in longer latency to the platform, indicating that spatial learning ability was declined in mice with PD, which is attenuated by FGF21 treatment (*P* < 0.001, Figures 2A,B). In the spatial probe test, MPTP injection reduces the number of crossing the place of the platform, which is also restored by FGF21 (*P* < 0.001, Figure 2C). The total distance in the target quadrant among different groups didn't show any significant alteration (*P* > 0.05, Figure 2D). In the Y-maze, the reduction in the spontaneous alternation induced by MPTP is also prevented by FGF21 (*P* < 0.001, Figure 2E).

### FGF21 Protected Against Neuronal Damage and Neuroinflammation in MPTP-Lesioned Mice

**Figure 3:**
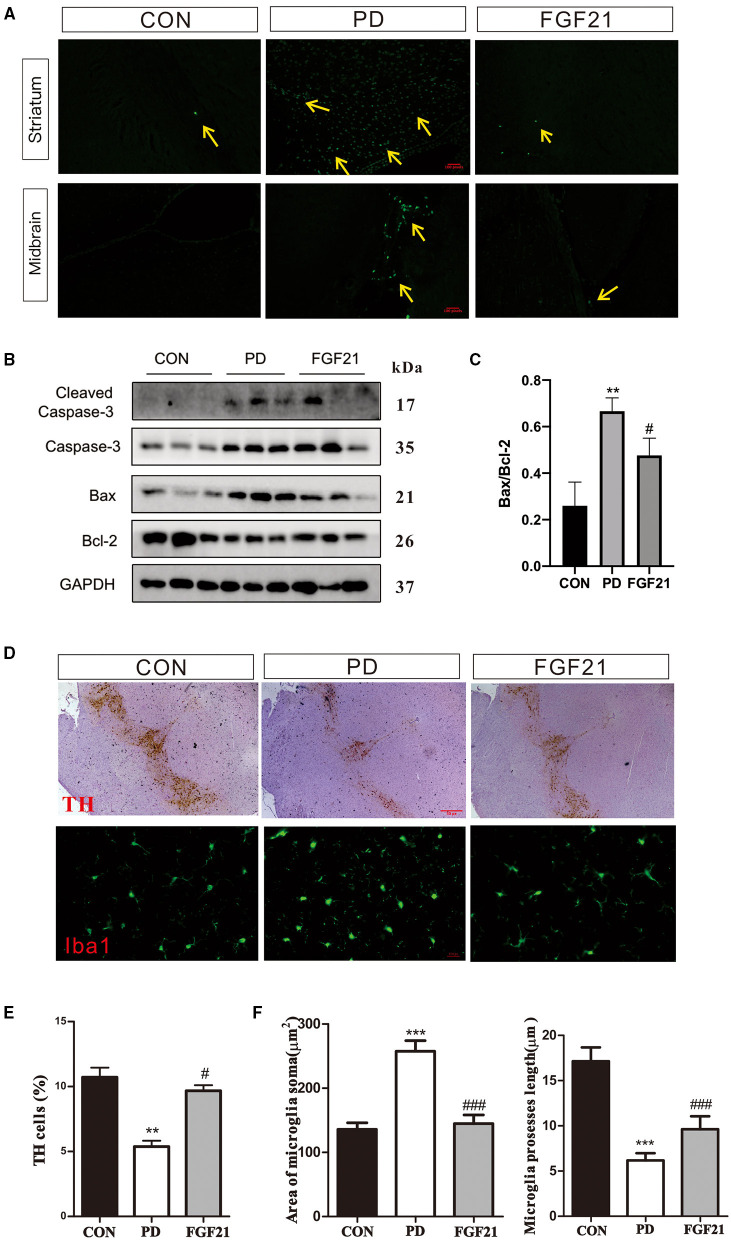
FGF21 treatment rescued PD-like pathology in mice with PD. **(A)** TUNEL assay. **(B)** Protein levels of Bcl-2, Bax, and cleaved caspase-3 in the midbrain. **(C)** Bcl-2/Bax ratio. **(D)** tyrosine hydroxylase (TH), and Iba-1 immunostaining. **(E)** TH-positive neurons in midbrain. **(F)** Microglia soma and microglia process length in midbrain. Data are mean ± s.e.m.; *n* = 12 for each group. A one-way ANOVA analysis was conducted followed by an LSD multiple comparison test. *P < 0.05, **P < 0.01, ***P < 0.001 vs. CON group; ^#^*P* < 0.05, ^##^*P* < 0.01, ^###^*P* < 0.001 vs. MPTP-injected mice.

Then, TUNEL and TH staining were carried out to investigate the effect of FGF21 treatment on dopamine neurons and apoptosis of nerve cells. FGF21 significantly prevented the neuronal apoptosis induced by MPTP in the midbrain and striatum (Figure 3A). Caspase-3, Bcl-2, and Bax are important factors directly related to neuronal cell apoptosis. Western blot analysis revealed that MPTP induced an increase in caspase-3 and Bax immunoreactivity, which was restored by FGF21 in the striatum (Figure 3B). In addition, the ratio of Bcl-2/Bax increased by FGF21, thus protecting against neuronal apoptosis in mice with PD (*P* < 0.001, Figure 3C). In addition, a significant reduction of TH-positive neurons was found in the midbrain of mice with PD, which is also prevented by FGF21 (*P* < 0.001, Figures 3D,E). In the midbrain of mice with PD, a great number of microglia was activated, as illustrated by Iba-1 immunofluorescence staining (*P* < 0.001, Figure 3D). FGF21 treatment significantly reduced Iba-1 immunoreactivity in the midbrain (*P* < 0.001, Figure 3D). The soma area and process length of microglia were also reversed after FGF21 treatment (*P* < 0.001, Figure 3F), further indicating that microglial activation may be involved in the protection of FGF 21 in PD.

### FGF21 Inhibited the NF-κB Pathway and Enhanced M2 Microglial Polarization in MPTP-Lesioned Mice

**Figure 4:**
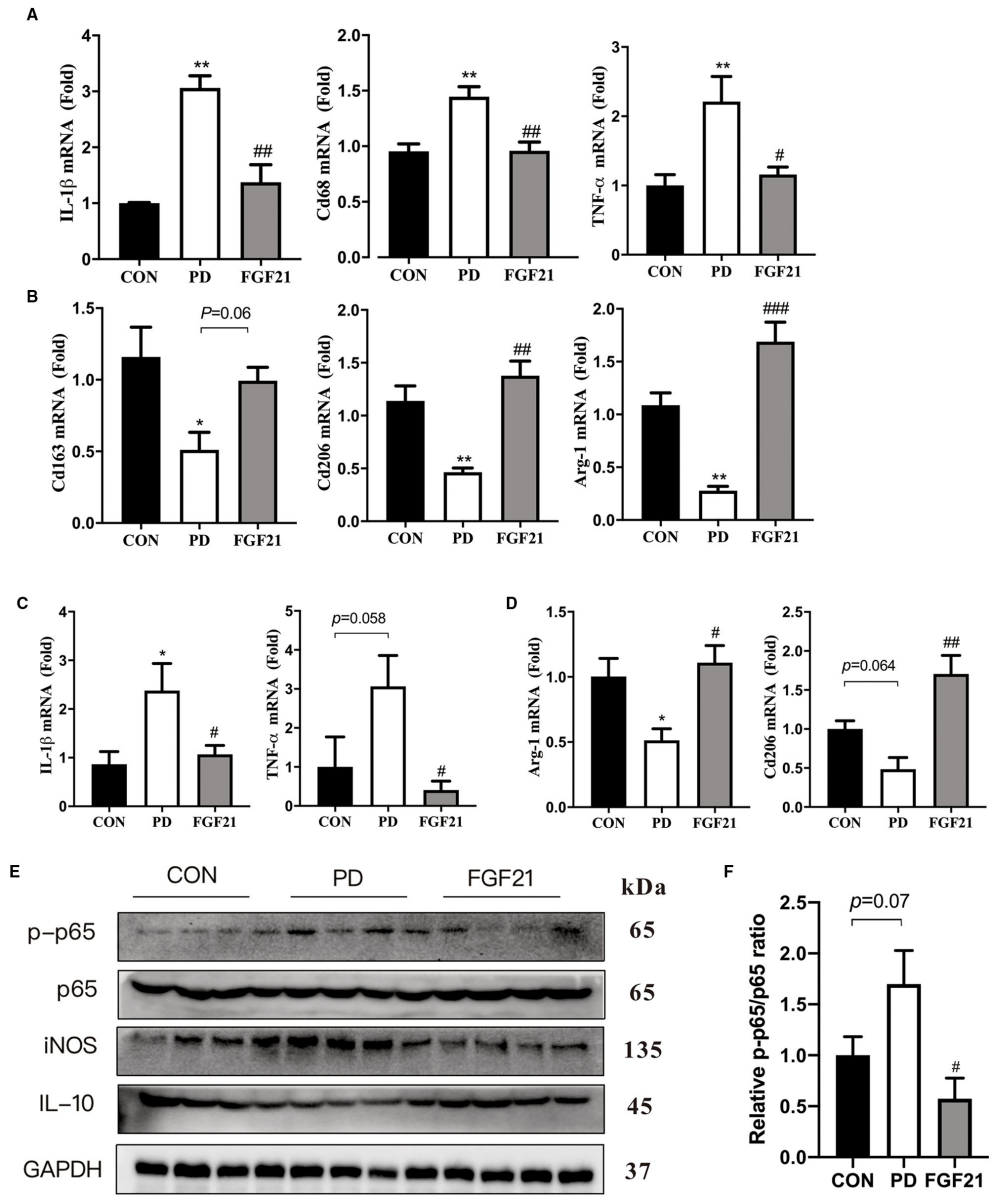
FGF21 inhibited the nuclear factor-kappa B (NF-κB) pathway and promotes M2 phenotypic transformation in the midbrain and striatum of mice with PD. qPCR analysis of microglial activation markers. M1 and M2 microglial genes in midbrain **(A,B)**. M1 and M2 microglial genes in striatum **(C,D)**. Data are mean ± s.e.m, *n* = 4 for each group. **(E)** Representative immunoblots of phosphorylated protein (p-p65), p65, iNOS, IL-10, and GAPDH in the ventral midbrain of CON, PD, and FGF21 mice. **(F)** Quantification of sirtuin 1 (SIRT1) and p-p65 levels in the ventral midbrain. Data are mean ± s.e.m, *n* = 4 for each group. A one-way ANOVA analysis was conducted followed by an LSD multiple comparison test. *P < 0.05, **P < 0.01 vs. CON group; ^#^*P* < 0.05, ^##^*P* < 0.01, ^###^*P* < 0.001 vs. MPTP-injected mice.

To further identify the role of microglial polarization in the protective effects of FGF21 in PD, the phenotype of microglia was detected in mice with PD with or without FGF21 treatment. Results showed that FGF21 significantly reduced the gene expression of IL-1β (*P* < 0.01), TNF-α (*P* < 0.01), and CD 68 (*P* < 0.05), the markers of M1 microglia (Figure 4A). Interestingly, MPTP injection reduces markers of M2 microglia (CD163, CD206, and Arg-1) in the midbrain, and this reduction is blocked by FGF21 (*P* < 0.05, Figure 4B). Similar results were found in the striatum, and the induction mRNA level of IL-1β and TNF-α is blocked by FGF21 treatment in mice with PD (*P* < 0.05, Figure 4C). More importantly, the upregulated levels of mRNA expression of M2 (CD206, Arg-1) genes in the striatum further confirmed that FGF21 promoted M2 phenotypic transformation in the midbrain and striatum (*P* < 0.05, Figure 4D). In addition, the phosphorylation of p65, a key member of the NF-κB pathway, was significantly elevated in mice with PD, and this activation was reduced by FGF21 (Figure 4E). Western blot analysis of iNOS and IL-10 that confirmed the activation of the NF-κB pathway by MPTP was inhibited by FGF21 treatment (Figure 4E). Quantitative analysis of Western blot results further suggested that FGF21 alleviates the microglia-mediated inflammatory response through inhibiting the NF-κB pathway (*P* < 0.05, Figure 4F).

### FGF21 Promoted the Transition From M1 to M2 Microglia by Elevating SIRT1 Activity

**Figure 5:**
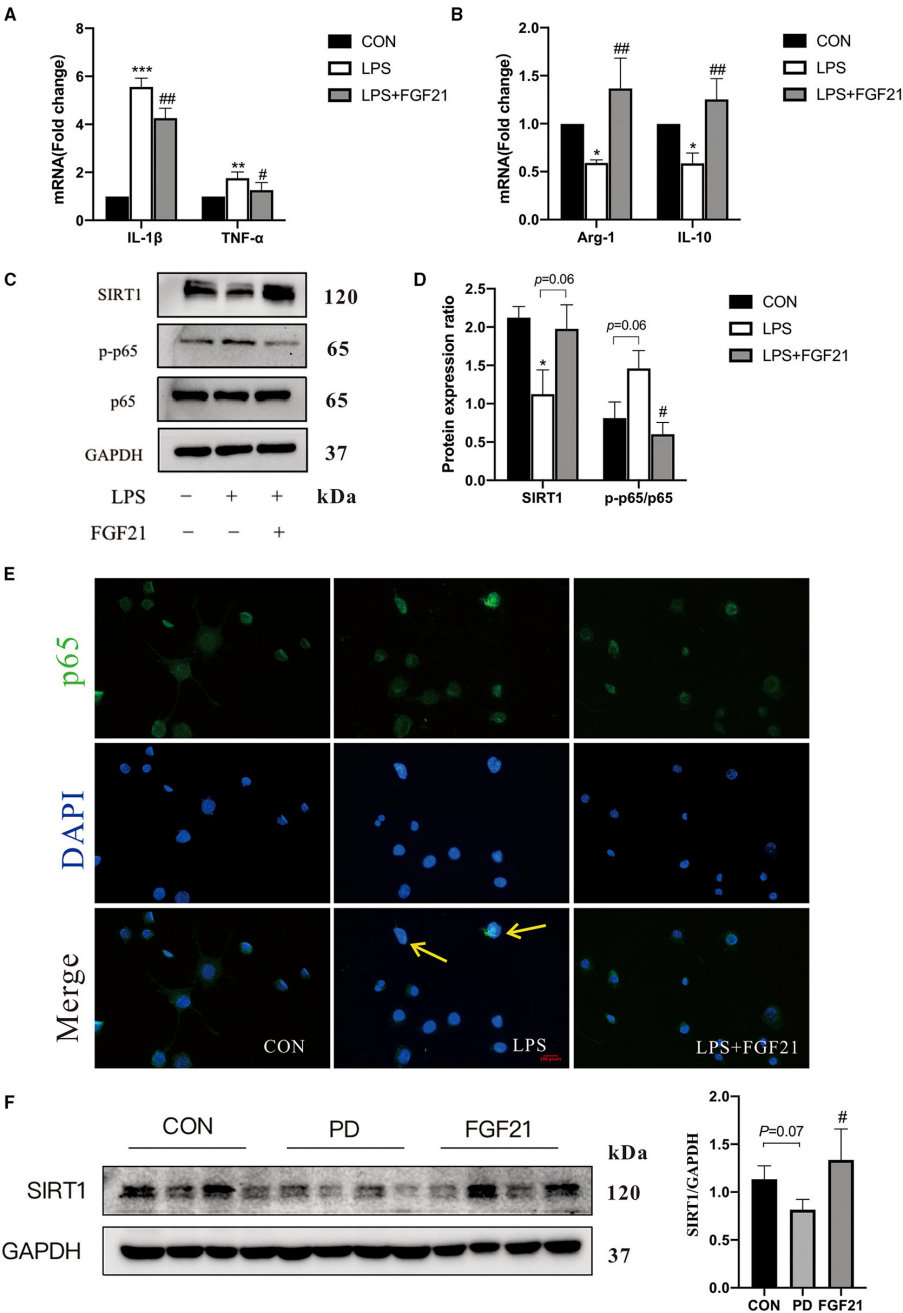
FGF21 inhibited LPS-induced microglial activation in BV2 cell cultures. Real-time PCR analysis of M1 and M2 gene markers in BV2 cells **(A,B)**. **(C)** Representative immunoblots of SIRT1, phosphorylated protein (p-p65), p65, and GAPDH in BV2 cells in response to LPS with or without FGF21 treatment (100 ng/ml). **(D)** Quantification of SIRT1 and p-p65 levels normalized to GAPDH. Data are mean ± s.e.m, *n* = 3 samples. **(E)** FGF21 inhibits translocation of NF-κB p65 from the cytosol to the nucleus. **(F)** Protein levels of SIRT 1 in the ventral midbrain of CON, PD, and FGF21 mice. A one-way ANOVA analysis was conducted followed by an LSD multiple comparison test. *P < 0.05, **P < 0.01, ***P < 0.001 vs. intact cells; ^#^*P* < 0.05, ^##^*P* < 0.01 vs. LPS-treated cells.

To further investigate the mechanisms underlying how FGF21 modulates microglial polarization, LPS-induced *in vitro* models with or without FGF21 treatment were conducted. The data revealed that the LPS-induced upregulation of IL-1β (*P* < 0.01) and TNF-α (*P* < 0.05) is blocked by FGF21 treatment in BV2 cell lines (Figure 5A). A significant increase in Arg-1 (*P* < 0.01) and IL-10 (*P* < 0.01) was seen in FGF21-treated cells (Figure 5B). In addition, protein levels of phosphorylation of p65 were significantly elevated in response to LPS, and this activation was also reduced by FGF21 (*P* < 0.05, Figures 5C,D). NF-κB p65 immunoreactivity staining further showed that the nucleus NF-κB p65 was significantly lower after FGF21 treatment, which revealed marker NF-κB inactivation after FGF21 treatment (Figure 5E). More importantly, SIRT1 protein, a deacetylase that influences NF-κB signaling and microglial activation, was upregulated by FGF21 treatment (*P* = 0.05, Figures 5C,D). Consistent with *in vitro* results, SIRT1 protein expression was also significantly increased by FGF21 treatment (*P* < 0.05, Figure 5F). The earlier results from both *in vitro* and *in vivo* proved that FGF21 may protect against microglial activation through SIRT1/NF-κB pathway in PD.

### Selective SIRT1 Antagonist Infusion Abolished FGF21 Effects in BV2 Cell Lines

**Figure 6:**
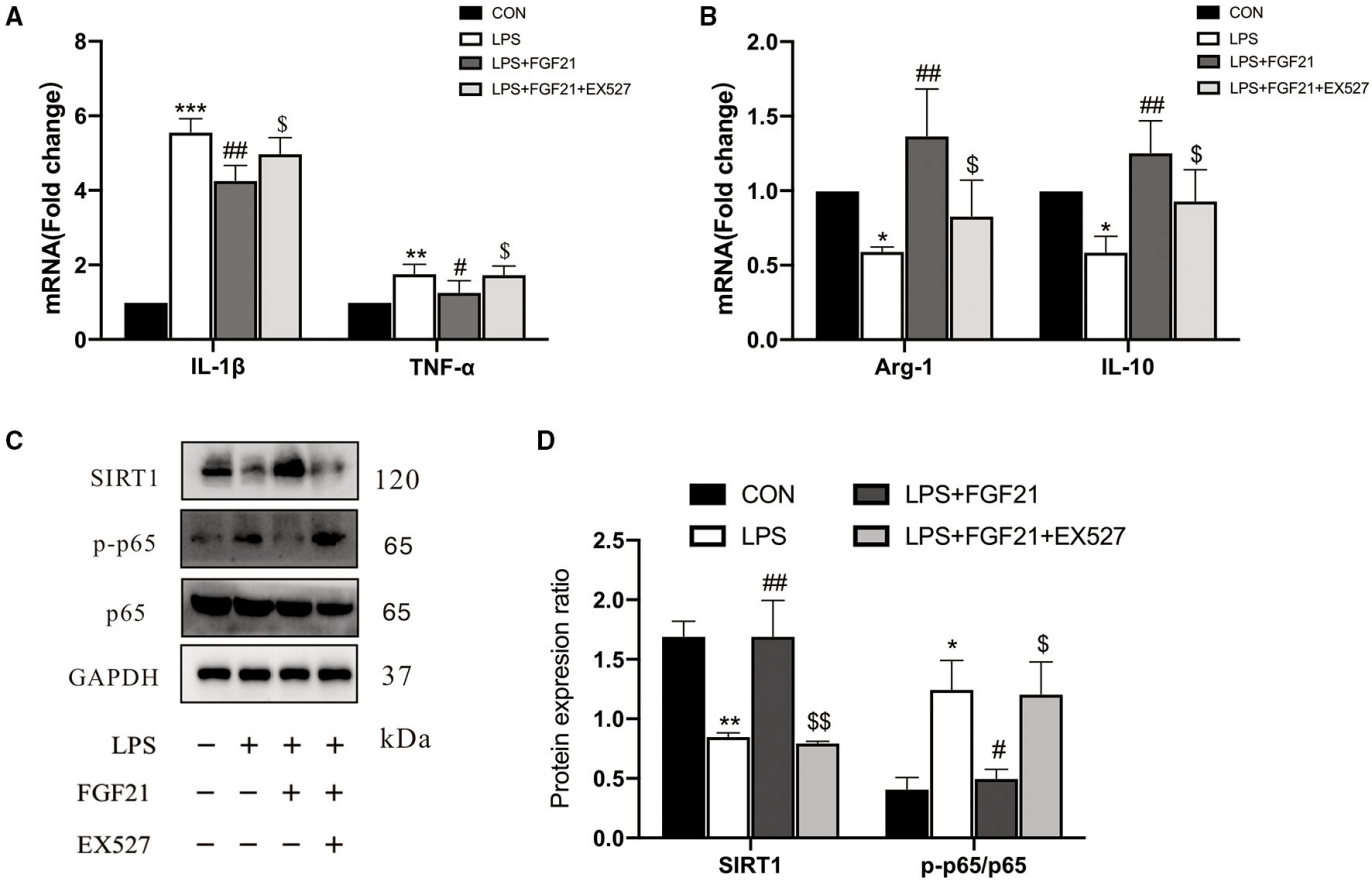
The inhibitory effects of FGF21 treatment on neuroinflammatory response were reversed by SIRT1 inhibitor (EX527). **(A,B)** Marker of M1 and M2 microglia in BV2 cells in response to FGF21 with or without SIRT1 inhibitor (EX527). **(C)** Representative immunoblots of SIRT1, phosphorylated protein (p-p65), p65, and GAPDH in BV2 cells in response to FGF21 with or without SIRT1 inhibitor (EX527). **(D)** Quantification of SIRT1 and p-p65 levels normalized to GAPDH. Data are mean ± s.e.m, *n* = 3 samples. A one-way ANOVA analysis was conducted followed by an LSD multiple comparison test. *P < 0.05, **P < 0.01, ***P < 0.001 vs. intact cells; ^#^*P* < 0.05, ^##^*P* < 0.01 vs. LPS-treated cells. ^$^*P* < 0.05, ^$$^*P* < 0.01 vs. FGF21+LPS-treated cells.

Based on the earlier data from *in vivo* and *in vitro*, it seems that SIRT1 may be involved in FGF21-mediated microglial polarization. We further focused on the role of SIRT1 activation, and the results showed that the anti-inflammatory action of FGF21 was blocked by SIRT1 inhibitor (EX527), as illustrated by the upregulation of IL-1β (*P* < 0.05) and TNF-α (*P* < 0.05) after adding SIRT1 inhibitor (Figure 6A). Similar results were found in the mRNA expression of Arg-1 and IL-10 (Figure 6B). Notably, the inhibitory effect of FGF21 on LPS-induced NF-κB p65 activation was also prevented by the selective SIRT1 inhibitor (*P* < 0.05, Figures 6C,D), indicating that FGF21-induced microglial M2 polarization was in part mediated by SIRT1.

### FGF21 Protects Against Dopaminergic Neuronal Loss Through Modulating Microglial Polarization

**Figure 7:**
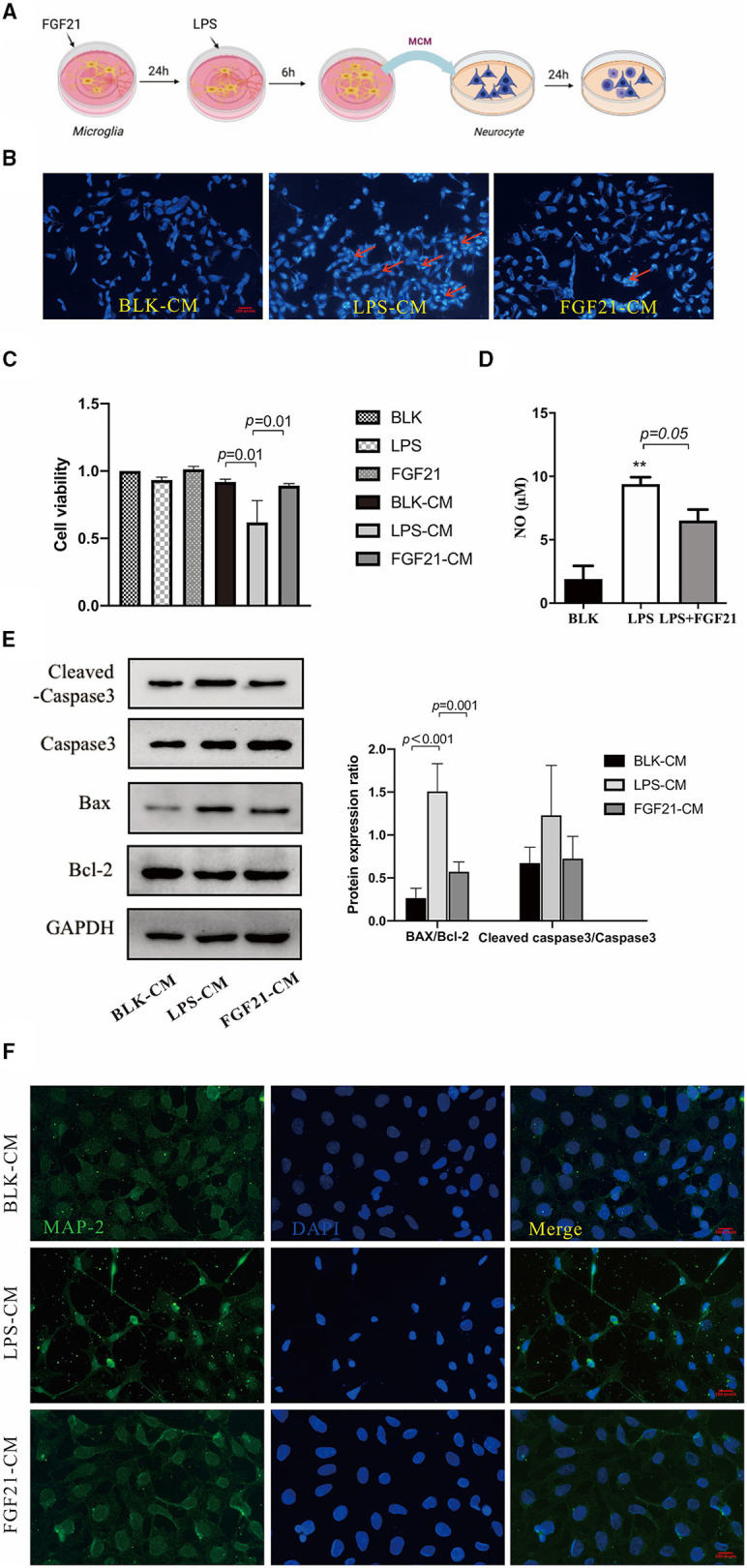
FGF21 protects against neuronal apoptosis in a microglia-dependent manner. **(A)** Treatment neurons with conditioned medium of microglia with or without FGF21 pre-treatment. **(B)** Representative images of Hoechst 33342 staining of SH-SY5Y treated with LPS-CM with vehicle or FGF21. Scale bar, 100 pixels. **(C)** Cell viability. **(D)** FGF21 pretreatment prevented the increase of NO in a microglia-conditioned medium. **(E)** MAP-2 (green) and DAPI (blue) immunostaining of SH-SY5Y cells. Scale bar, 100 pixels. **(F)** expression levels of Bcl-2, Bax, and cleaved caspase-3 in SH-SY5Y cells. Data are mean ± s.e.m, *n* = 3 samples. A one-way ANOVA data analysis was conducted followed by an LSD multiple comparison test. **P < 0.01 vs. intact cells.

To ascertain whether microglial polarization is responsible for the neuroprotective effects of FGF21, a microglial-conditioned medium from LPS-treated BV2 cells was applied to SH-SY5Y neuroblastoma cells. Neuronal apoptosis was determined using Hoechst 33342 staining (Figure 7A). Representative images of Hoechst 33342 staining were shown in Figure 7B, LPS-CM induces neuronal apoptosis (red arrows), and this induction is blocked by FGF21. Further cell viability analysis confirmed the protective effects of FGF21 (*P* < 0.01, Figure 7C). Cell apoptosis markers, including caspase-3, Bcl-2, and Bax, were significantly increased after LPS-CM incubation, which is also restored by FGF21 (*P* < 0.001, Figure 7E). Besides, in SH-SY5Y cell cultures, direct treatment of FGF21 failed to protect against LPS-CM-induced cell death (Figure 7C), indicating that the action of FGF21 is likely to be mediated by microglial cells. The NO released by LPS stimulation is significantly inhibited by FGF21 (Figure 7D). Notably, the treatment of LPS-CM significantly reduced the MAP-2 immunoreactivity, while this reduction is blocked by FGF21 (Figure 7F), indicating that FGF21 protects against dopamine neuronal apoptosis indirectly through the inhibition of microglial activation.

## Discussion

**Figure 8:**
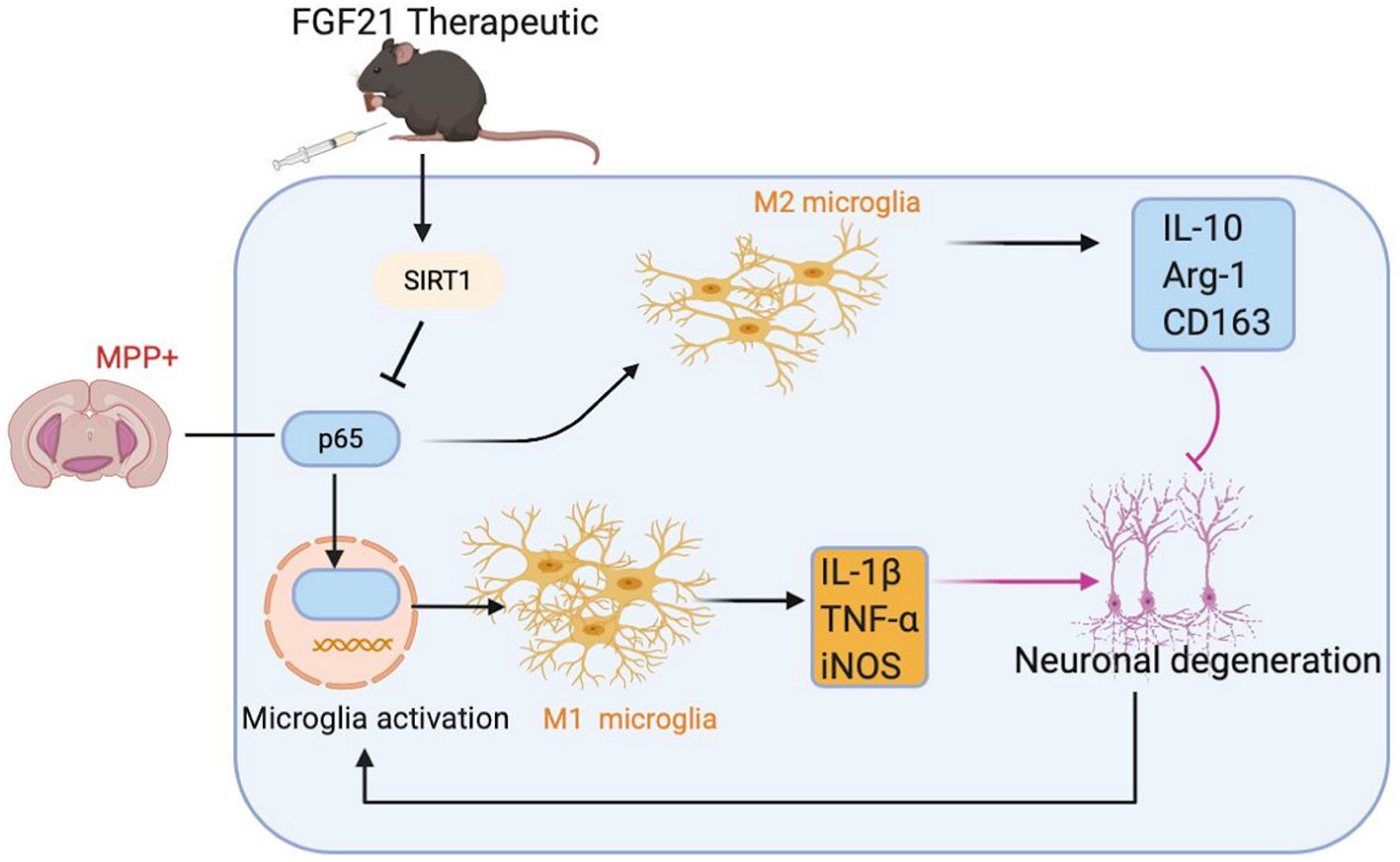
Potential molecular mechanisms of FGF21 in treatment of PD *via* SIRT1/NF-κB pathway-mediated microglial polarization. MPTP injection activates NF-κB signaling in microglia, resulting in the microglial activation to assume an “M1” -like phenotype, producing pro-inflammatory cytokines, such as IL-1β and TNF-α, which contributes to the death of dopaminergic neurons in MPTP-induced PD mice model and in SH-SY5Y neuronal cultures. FGF21 treatment inhibits NF-κB signaling in microglia through upregulating SIRT1 expression, transforming activated M1 microglia to M2 microglia. M2 microglia then produces anti-inflammatory factors, such as IL-10, which result in strong neuroprotective effects in PD.

In this study, we illustrated for the first time that FGF21 remarkably attenuated motor symptoms and cognitive dysfunctions in mice induced by MPTP. Most importantly, the protective effects of FGF21 are independent of its metabolic regulating role on dopaminergic neurons but appear to be mediated through microglial polarization. We showed that MPTP induces a sustained M1 microglial activation, while FGF21 enhances the M1/M2 microglial polarization and suppresses the microglia-mediated inflammatory response in mice with PD. Both *in vivo* and *in vitro* results further confirmed that FGF21 promotes M2 microglial polarization through modulating SIRT1/NF-κB pathway. This M2 microglial polarization then contributed to the protective role of FGF21 in SH-SY5Y neuronal cultures (Figure 8). Results from this study provide novel molecular mechanisms on how FGF21 exerted its protective effects in the pathology of PD through SIRT1/NF-κB pathway-mediated microglial polarization.

Non-motor features such as sleep disorders, olfactory impairment, and cognitive dysfunction may arise before the clinical motor symptoms (Marinus et al., 2018). MPTP-induced mice have been shown to display irreversible PD-like behavioral impairments and microglial activation (Jackson-Lewis and Przedborski, 2007; Lee et al., 2019). In this study, we used this valid model to investigate the effects of FGF21 on motor and non-motor symptoms in PD and to clarify its underlying molecular mechanisms. Our results confirmed that FGF21 treatment significantly alleviated the motor impairment in MPTP-induced PD model, as shown in the pole test and rotarod test. Early dopaminergic neuronal death in the substantia nigra pars compacta is the characteristic feature of PD (Kalia and Lang, 2015). Accordingly, FGF21 significantly prevented the loss of TH-positive neurons in the midbrain of MPTP-injected mice. More importantly, results from the Y-maze and Morris water maze, which are widely used to evaluate cognitive function (Lainiola et al., 2014) and spatial memory (Nunez, 2008), showed that FGF21 also markedly prevented the MPTP-induced cognitive impairment in mice with PD. An important limitation of this study is that it was performed in a subacute MPTP mice model of PD. Further clinical and basic research on the long-term protective effects of FGF21 should be encouraged to validate the use of this drug.

Microglial activation and the subsequent neuroinflammation have recently been confirmed to play multifarious roles in the dopaminergic neuronal degeneration in PD, with increased concentrations of pro-inflammatory cytokines in the blood and brain of patients with PD (Nagatsu et al., 2000; Gersel Stokholm et al., 2020). In this study, we found that FGF21 significantly reduced the Iba-1 immunoreactivity in the midbrain, suggesting that FGF21 could inhibit microglial activation in mice with PD. Meanwhile, alpha-synuclein pathology plays a very important role in PD progression (Rai et al., 2017; Zahra et al., 2020), and increasing evidence suggests that alpha-synuclein invasion could activate M1 microglia, which then promote the transmission of alpha-synuclein to neuronal cells *via* exosomal pathways (Xia et al., 2019). More recent studies identified that microglia accumulation of alpha-synuclein could contribute to the primary event in dopaminergic neurodegeneration through the production of oxidative and pro-inflammatory molecules (Bido et al., 2021). Collectively, FGF21-induced microglia inactivation might contribute to the neuroprotective effects of FGF21 in PD.

Previous studies have shown that the release of various pro-inflammatory factors by activated M1 microglia, such as IL-1β and NO, will cause obvious biological damage to neuronal cells, and the inhibition of their expression is neuroprotective in PD models (Sanchez-Guajardo et al., 2013; Xia et al., 2019). Cheng et al. found that the low inflammatory state induced by FGF21 would contribute to its protective effects in PD (Fang et al., 2020). In agreement with the previous finding, we found that gene expression of pro-inflammatory M1 factors such as IL-1β, TNF-α, and CD68 was downregulated by FGF21 in MPTP-lesioned mice. Other studies have shown that M2 polarization may exert neuroprotective effects in animal models of PD (Park et al., 2016; Calvello et al., 2017; Tamtaji et al., 2018). In particular, M2-polarized microglia has been proved to dampen pro-inflammatory immune reactivity by producing anti-inflammatory cytokines, such as IL-4, IL-10, and transforming growth factor beta (TGF-β) (Park et al., 2016). In this study, the mRNA level of anti-inflammatory M2 molecules, including IL-10, CD163, CD206, and Arg-1, was significantly upregulated by FGF21 treatment and, most notably, IL-10 itself has been identified to promote M1/M2 transition (Deng et al., 2012), suggesting a shift from M1 pro-inflammatory phenotype to M2 anti-inflammatory phenotype. More importantly, microglial-conditioned medium from LPS-induced BV2 cells significantly induced neuronal apoptosis in dopaminergic SH-SY5Y cells. These results suggested that FGF21 treatment of BV2 cells, but not neuronal cells, prevented the production of NO and neuronal cell death. The earlier results identified that the transition of microglial M1/M2 polarization is primarily involved in the protective effects of FGF21 in PD.

The NF-κB pathway has long been identified to modulate inflammatory responses and iNOS in activated microglia cells (Park et al., 2015). Here, we found that the translocation of NF-κB p65 into the nucleus was retarded by FGF21 treatment, thus could attenuate NF-κB p65 activation both *in vivo* and *in vitro*. Previous studies identified that SIRT1 is downregulated in PD, and upregulating SIRT1 is neuroprotective against MPTP-mediated neurotoxicity (Singh et al., 2017). Of interest, more recent studies have shown that the effects of FGF21 are highly related to SIRT1 expression (Chen et al., 2021), and SIRT promoted FGF21 sensitivity neuronal cells (Matsui et al., 2018). We then examined the SIRT1 expression in PD models, and our results showed that FGF21 significantly upregulated the SIRT1 expression and inhibited the activation of the NF-κB pathway both *in vivo* and *in vitro*. Furthermore, the anti-inflammatory effects of FGF21 are diminished by a selective SIRT1 inhibitor, suggesting that the effects of FGF21 on microglial polarization are mediated at least partially by SIRT1/NF-κB pathway. To the best of our knowledge, this study was the first report of the polarizing effect on microglia of FGF21 both *in vivo* and *in vitro* co-culture models of PD.

This study provides both *in vivo* and *in vitro* evidence that FGF21 potently protects the dopaminergic neurons through the promotion of microglial M2 polarization *via* the SIRT1/NF-κB pathway in a PD model. Unlike conventional therapies that directly improve dopaminergic neurons, the neuroprotective role of FGF21 in PD is mediated indirectly through modulating M1/M2 microglial polarization. Considering the safety record of human clinical trials, FGF21 could be a promising therapy for clinical trials to ameliorate motor and non-motor deficits in patients with PD.

## Data Availability Statement

The raw data supporting the conclusions of this article will be made available by the authors, without undue reservation.

## Ethics Statement

The animal study was reviewed and approved by Institutional Animal Care and Use Committee of Wenzhou Medical University.

## Author Contributions

CY: conceptualization, writing—original draft, and founding acquisition. WW: investigation and methodology. PD: visualization and investigation. CL: data duration and supervision. LZ: resources. HG: writing—review and editing, and funding acquisition. All authors have read and approved the final manuscript.

## Funding

This work was supported by the National Natural Science Foundation of China (Grant Nos. 21974096 and 81771386) and the Natural Science Foundation of Zhejiang Province (Grant No. LQ19H070001).

## Conflict of Interest

The authors declare that the research was conducted in the absence of any commercial or financial relationships that could be construed as a potential conflict of interest.

## Publisher's Note

All claims expressed in this article are solely those of the authors and do not necessarily represent those of their affiliated organizations, or those of the publisher, the editors and the reviewers. Any product that may be evaluated in this article, or claim that may be made by its manufacturer, is not guaranteed or endorsed by the publisher.

## Abbreviations

MPTP, 1-Methyl-4-phenyl-1, 2, 3: 6-tetrahydropyridine
PD: Parkinson's disease
FGF21: fibroblast growth factor-21
CON: control
TH: tyrosine hydroxylase
iNOS: inducible nitric oxide synthase
SIRT1: sirtuin 1
NF-κB: nuclear factor-kappa B
TNF-α: tumor necrosis factor
IL-1β: interleukin 1beta
NO: nitric oxide
Arg-1: arginine
LPS: lipopolysaccharide
CM: conditioned medium
CCK-8: Cell Counting Kit-8
MAP-2: microtubule-associated protein 2
TGF-β: transforming growth factor beta
SDS-PAGE: sodium dodecyl sulfate polyacrylamide gel electrophoresis
PVDF: polyvinylidene fluoride.
