# Supplementary material for: Fibroblast Growth Factor 21 Modulates Microglial Polarization That Attenuates Neurodegeneration in Mice and Cellular Models of Parkinson's Disease
Source: Front Aging Neurosci. 2021 Dec 22;13:778527. doi: 10.3389/fnagi.2021.778527 (PMC8727910; doi:10.3389/fnagi.2021.778527)
Supplement: Supplementary file 1 [file Image_1.pdf]

Supplemental Material

Fibroblast growth factor 21 modulates microglial polarization that attenuates neurodegeneration in mice and cellular models of Parkinson's disease

Changwei Yang[1, 2#], Wuqiong Wang[1#], Pengxi Deng[1], Chen Li[1], Liangcai Zhao[1], Hongchang Gao[1]*

[1] Institute of Metabonomics & Medical NMR, School of Pharmaceutical Science, Wenzhou Medical University, Wenzhou 325035, China;

[2] School of Public health, Fujian Medical University, Fuzhou 350122, China;

[#] Changwei Yang and Wuqiong Wang contributed equally to this work.

[*]Correspondence:

Hongchang Gao

gaohc27@wmu.edu.cn (H.C. Gao);

Running title: FGF21 attenuates neurodegeneration in PD

Figure list

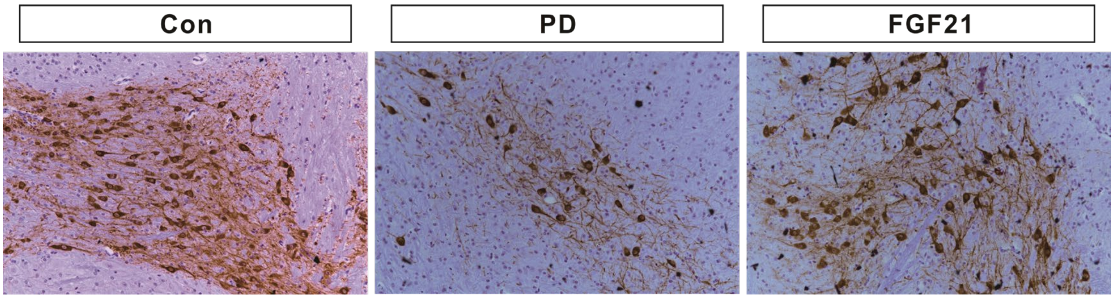

Fig S1 High-magnification (400X) images of immunohistochemical staining for Tyrosine hydroxylase (TH) in the midbrain
